# Migraine in Multiple Sclerosis Patients Affects Functional Connectivity of the Brain Circuitry Involved in Pain Processing

**DOI:** 10.3389/fneur.2021.690300

**Published:** 2021-08-12

**Authors:** Emanuele Pravatà, Gianna C. Riccitelli, Carlo Sestieri, Rosaria Sacco, Alessandro Cianfoni, Claudio Gobbi, Chiara Zecca

**Affiliations:** ^1^Neuroradiology, Neurocenter of Southern Switzerland, Ospedale Regionale di Lugano Civico e Italiano, Lugano, Switzerland; ^2^Headache Center, Neurocenter of Southern Switzerland, Ospedale Regionale di Lugano Civico e Italiano, Lugano, Switzerland; ^3^Department of Neurology, Neuropsychology and Behavioural Neurology Research Unit, Neurocenter of Southern Switzerland, Ospedale Regionale di Lugano Civico e Italiano, Lugano, Switzerland; ^4^Department of Neuroscience, Imaging and Clinical Sciences, Gabriele D'Annunzio University of Chieti and Pescara, Chieti, Italy; ^5^Faculty of Biomedical Sciences, Università della Svizzera Italiana, Lugano, Switzerland

**Keywords:** multiple sclerosis, migraine, pain, functional magnetic resonance, functional connectivity

## Abstract

Migraine is particularly common in patients with multiple sclerosis (MS) and has been linked to the dysfunction of the brain circuitry modulating the peripheral nociceptive stimuli. Using MRI, we explored whether changes in the resting state-functional connectivity (RS-FC) may characterize the occurrence of migraine in patients with MS. The RS-FC characteristics in concerned brain regions were explored in 20 MS patients with migraine (MS+M) during the interictal phase, and compared with 19 MS patients without migraine (MS-M), which served as a control group. Functional differences were correlated to the frequency and severity of previous migraine attacks, and with the resulting impact on daily activities. In MS+M, the loss of periaqueductal gray matter (PAG) positive connectivity with the default mode network and the left posterior cranial pons was associated with an increase of migraine attacks frequency. In contrast, the loss of PAG negative connectivity with sensorimotor and visual network was linked to migraine symptom severity and related daily activities impact. Finally, a PAG negative connection was established with the prefrontal executive control network. Migraine in MS+M patients and its impact on daily activities, underlies RS-FC rearrangements between brain regions involved in pain perception and modulation.

## Introduction

Migraine affects up to 43% of patients with multiple sclerosis (MS), with a significantly higher prevalence than in the general population (1). In MS-unrelated migraine, clinical and electrophysiology studies have shown that the development of attacks is linked to the dysfunctions in the interaction between areas of the brain network deputed to pain stimuli palliation and processing (2, 3). In this circuit, the periaqueductal gray matter (PAG) has a pivotal role in the modulation of peripheral hyperexcitability developing along the trigemino-vascular system (2, 3). Understanding neurophysiological changes occurring between the PAG and its connected brain regions in patients with MS could be important to develop future specific pain intervention strategies, such as non-invasive transcranial stimulation (4, 5). To the best of our knowledge, there are few previous studies in the literature which specifically assessed the occurrence of PAG demyelinating lesions in MS patients with migraine, and the relationship between such lesions and patients' symptoms severity remains controversial. Indeed, whereas a previous study showed an increased risk of lesion occurrence in migraineurs MS patients (6), two others did not find any relationship with migraine symptoms ((7, 8); see also (9) for a review). The spontaneous functional MRI dynamics occurring *between* brain regions can be assessed using the resting-state functional connectivity (RS-FC) (10) technique. In healthy subjects, RS-FC identified the brain regions exhibiting significant connectivity with the PAG (11, 12). These regions encompassed the so-called pain modulatory network (PMN) (12). Notably, the PAG exhibited either positive or negative connectivity with other brain regions (11, 12), respectively indicating functional integration or segregation (13, 14).

The aim of the present work was to investigate the neural base of the occurrence of migraine in MS patients. Specifically, we tested whether RS-FC modifications of the PAG, and the connected pain processing circuitry regions, may characterize the occurrence of migraine in patients with MS. Because MS disease exerts *per se* a large and widespread impact on the brain functional organization {(15) #287; (16) #288}, in order to specifically capture the effect of migraine occurrence, we assessed functional differences in MS patients with migraine (MS+M) against a group of MS patients without migraine (MS-M) who were matched for their demographic, neurological, neuropsychological, education, and treatment characteristics. We tested (1) whether the presence of migraine affects the connectivity between the PAG and regions that are either positively and/or negatively connected, and (2) whether migraine symptoms are associated with any of the encountered changes.

## Materials and Methods

### Participants

Twenty MS+M and 19 matched MS-M patients were consecutively enrolled at the MS center of Civic Hospital in Lugano (Ticino, Switzerland). All patients were right-handed, between 20 and 55 years old, with relapsing-remitting MS according to McDonald criteria (17). They were relapse- and steroid-free for at least 3 months and were not on any analgesic medication at the time of the study.

Patients with migraine had to fulfill additional inclusion criteria: (1) diagnosis of episodic migraine with at least 1 attack per month or chronic migraine according to the International Headache Society criteria (18); (2) no acute migraine attacks within the 72 h prior to the scanning session; (3) no treatment with psychotropic medications, like antidepressant and/or antiepileptic agents; (4) no migraine prophylactic treatments. Patients with significant medical illness or substance abuse that could have interfered with cognitive functioning, other major systemic, psychiatric or neurological diseases, and history of alcohol/drug abuse were excluded.

### Clinical Assessment

On the day of MRI acquisition all patients underwent Expand Disability Status Scale (EDSS) assessment. In MS+M, migraine disease duration and the number of monthly migraine days (MMD) during the last month according to patient's migraine diary were captured. The patients were asked to indicate average pain intensity during migraine attacks of the last 2 months on a 10 cm visual analog scale (VAS-P) (0 no pain, 10 maximum unbearable pain). They completed the six-item Headache Impact Test (HIT-6) (19) to assess grade of impact on daily life activity. Within 48 h after MRI acquisition, all patients attended a semi-structured interview investigating depression and anxiety symptoms using the Hamilton depression rating scale (HDRS) (20) and Hamilton anxiety rating scale (HAM-A) (21). All patients were relapse- and steroid-free for at least 3 months, with unchanged MS modifying treatment during 6 months before MRI acquisition.

### MRI Data Acquisition

All data were acquired on a single 3.0T scanner (Siemens Skyra, Erlangen, Germany) using a 20-channel head coil. Anatomical images consisted of Magnetization-Prepared-Rapid-Gradient-Echo (MPRAGE) 3D T1-weighted (TR = 1,900 ms; TE = 2.1 ms; TI = 900 ms; FOV = 240 mm^2^; matrix = 256 × 256; voxel size = 0.9 × 0.9 × 0.9 mm^3^), and 3D Dark-Fluid T2-weighted (TR = 5,000 ms; TE = 394 ms; TI = 1,800 ms; FOV = 240 × 240 mm^2^; matrix = 256 × 256; voxel size = 0.9 × 0.9 × 0.9 mm^3^) sequences. RS-FC data were obtained by using a BOLD single-shot echo-planar sequence (TR = 1,800 ms; TE = 30 ms; flip angle = 90°; FOV = 240 mm^2^; matrix = 64 × 64; slices number = 32; slice thickness = 4 mm; gap = 0; voxel size = 3.75 × 3.75 × 4 mm; volumes = 230; acceleration factor = 2; scan duration = 6'54”). During rs-fMRI data acquisition, participants were asked to keep their eyes closed. As we used a multichannel coil and focused our analyses on the PAG, a deeply-located brainstem structure, pre-scan signal intensity normalization was applied to optimize signal homogeneity (22).

### Structural Data Analysis

T_2_-visible lesions were automatically segmented on the 3D-Dark-Fluid images using Lesion Segmentation Toolbox (LST) (http://www.statistical-modeling.de/lst.html). Subsequent manual refinement was performed by an experienced neuroradiologist (E.P.) using MRIcron (https://www.nitrc.org/projects/mricron). The resulting volume (T_2_-LV) was recorded for each patient. The corresponding segmentations were used to refill T1-weighted images with voxels of similar signal intensity to that of the adjacent white matter (WM) before these were submitted to further analyses, in order to reduce tissue segmentation bias. Normalized brain volume (NBV), gray matter (GM) volume (NGMV), and WM volume (NWMV) were estimated on 3D-MPRAGE images, using Structural Imaging Evaluation of Normalized Atrophy—cross-sectional (SIENAx), part of FSL. Finally, T2-lesion probability maps (T_2_-LPM) were generated by voxel-wise comparison of the lesions spatial frequency, using the non-parametric Liebermeister test (23) with “non-parametric mapping” (NPM-www.mricro.com).

### Functional Data Preparation

All BOLD analyses were conducted with the Functional Connectivity toolbox (CONN) v17f (https://www.nitrc.org/projects/conn) (24). Because the standard regression of the whole brain signal, a preprocessing option used to remove noise, may bias the estimation of the negative correlations (25), we employed the software embedded CompCor tool instead (26). This corrects for physiological noise by regressing out the principal components from the white matter and cerebral spinal fluid, where the signal is unlikely related to neural activity. Time-series images were realigned, slice timing corrected, normalized into the Montreal Neurological Institute (MNI) standard space, and spatially-smoothed with a 6 mm FWHM filter to improve between-subjects comparability. GM, WM, and cerebrospinal fluid segmentations maps were obtained. Subject head motion confound was accounted using functional outlier detection, by running the Artifact Detection Tools (ART, https://www.nitrc.org/projects/artifact_detect) based algorithm in CONN. Outlier volumes were identified *via* framewise displacement using default parameters (>0.9 mm frame-wise displacement), and then regressed out. Furthermore, by using CompCor, the estimated subject-motion parameters (three translation, three rotation plus their associated first-order derivatives) were regressed out from the BOLD time series. Time courses were de-trended and filtered (0.01–0.08 Hz) to retain the low frequency fluctuations (LFF) range.

### PAG Connectivity Analysis

All RS bold signal analyses were restricted to the GM by employing the previously generated tissue masks. By employing previously described anatomical and functional descriptions of the PAG as references (27, 28), a 3 mm radius spherical ROI was visually drawn at the level of the PAG on the high-resolution T1-weighted image of each patient (Supplementary Figure 1). Seeds were subsequently transferred into the MNI standard space and served as origins for the seed-to-voxel analyses. Seed-based connectivity maps were calculated using Fisher-transformed bivariate correlation coefficients, with time series being centered to zero mean. All cluster-level inferences were conducted with parametric statistics performed using CONN. One-sample *t*-test statistics are then performed, with the positive and negative directionality reflecting the positive and negative correlation sign against zero. Differences between groups were assessed with two-sample *t*-tests, with positive and negative contrasts conducted separately corresponding to one-side comparison. Analyses were adjusted for age and gender. The employed cluster height significance threshold was *P* < 0.01 uncorrected, with a cluster-size level threshold of *P* < 0.05 corrected with false discovery rate (FDR).

In order to combine the voxel-wise analysis performed in our specific groups of patients with a regions-of-interest approach using ROIs taken from the literature, we used the regions identified by Shirer et al. because (i) they derive from a data-driven approach, (ii) they are freely available, and (iii) they have been employed in several previous studies (29, 30). While we initially performed analyses in ROIs from all the available networks, we subsequently decided to focus on those networks that overlap with clusters of PAG connectivity. In order to determine the PAG connectivity relationships with respect to the main intrinsic connectivity brain networks (ICNs), the resulting clusters were overlaid on the ICNs anatomical maps provided in (31). These include the default mode network (DMN), the basal ganglia network (BGN), sensorimotor network (SMN), higher visual network (HVN), auditory network (AN), executive control network (ECN), and posterior salience network (PSN) (Supplementary Figure 2).

For each comparison, all clusters overlapping on one single ICN were averaged together and the resulting functionally defined region-of-interest (fROI) was named with that particular ICN.

Clusters without ICN overlap were named according to their specific anatomical peak. For each of the resulting fROI, average RS-FC values were extracted using REX (https://www.nitrc.org/projects/rex/) for regional analyses.

Finally, patients' migraine VAS, HIT-6, and MMD scores were correlated with the average RS-FC values extracted from the clusters obtained in the seed-to-voxel between-groups comparison map.

### Additional Statistical Analysis

The Shapiro-Wilk test was used to verify normal distribution of demographic data, clinical variables, and structural MRI measurements. Chi-square test or Fisher's exact test and Mann–Whitney *U*-test were applied to investigate between-group differences, Spearman's Rank-Order Correlation to test associations between clinical and RS-FC measurements. Significance level was set at *P* < 0.05 after Bonferroni correction for multiple comparisons. Data were analyzed using the statistical software package SPSS (version 25.0).

## Results

### Demographic, Clinical, and Structural MRI Characteristics

Table 1 summarizes the main demographic, clinical, and MRI structural characteristics of the patients. The two study groups were matched for gender, age, disability, and MS-related disease duration. The proportion of patients on MS disease modifying treatment (*p* = 0.65) as well as anxiety and depression symptoms severity were similar between groups (respectively *p* = 0.38 and *p* = 0.36).

**Table 1:** Main demographic, clinical, and structural MRI characteristics in MS+M and MS-M patients.

	**MS+M**	**MS-M**	***P***
*N*	20	19	
Men/Women	3/17	3/16	0.27**^$^**
Median age (IQR)	39.8 (30–47.3)	43.2 (35–46.9)	0.59^#^
Median EDSS (IQR)	2.5 (2–3)	2.5 (2–3)	0.77^#^
Median MS DD (yrs) (IQR)	6.5 (2–9)	8.0 (4–12)	0.21^#^
Median migraine DD (yrs) (IQR)	4.5 (2–17)	-	-
Frequencies of patients on DMT for MS (oral, injectable, infusion)	20 (30%; 45%; 25%)	19 (42%; 32%; 26%)	0.65^*^
Median HAM (IQR)	13 (7–18)	11 (3–17)	0.38^#^
Media HDRS	8 (4–15)	10 (6–15)	0.36^#^
Number of MMD (day /mo.) (IQR)	6 (3–8)	-	-
Median VAS-P (IQR)	7 (6–8)		
Median HIT-6 (IQR)	62 (57–68)	-	-
Median T_2_ lesion volume ml (IQR)	3.3 (3.0–3.8)	3.1 (2.8–3.6)	0.66^#^
Median NBV ml (IQR)	1,514 (1,421–1,593)	1,501 (1,442–1,551)	0.31^#^
Median NWMV ml (IQR)	713 (663–762)	686 (672–716)	0.31^#^
Median NGMV ml (IQR)	799 (750–840)	802 (739–831)	0.96^#^

*MRI, Magnetic Resonance Imaging; MS+M, Multiple sclerosis patients with migraine; MS-M, Multiple sclerosis patients without migraine; IQR, Interquartile range; EDSS, Expand Disability Status Scale; DD, Disease duration; MMD, Monthly migraine days; VAS-P, Visual analog scale; HIT-6, Six-item headache impact; HAM-A, Hamilton anxiety symptoms; HDRS, Hamilton depression rating scale; NBV, Normalized brain volume; NWMV, Normalized white matter volume; NGMV, Normalized gray matter volume*.

$ *Fisher's exact Test;*

* *Chi-square Test;*

# *Mann-Whitney U-Test*.

Also, no significant between-group difference was detected for normalized brain, GM, WM, overall T_2_ lesion volume, and spatial distribution (images not shown).

MS+M patients had an average migraine disease duration of 4.5 (IQR = 2–17) years, six (30%) had migraine with aura who did not differ for demographic, clinical, and structural parameters with respect to patients without aura; *p* = 0.74. The median HIT-6 score was 62 (IQR = 57–68), MMD 6 (IQR = 3–8) days, and VAS-P score 7 (IQR = 6–8). MMD was not significantly correlated with HIT-6 (rho = −0.246, *p* = 0.148) and VAS-P scores (rho = −0.232, *p* = 0.162), whereas the HIT-6 scores were significantly correlated with VAS-P scores (rho = 0.374, *p* = 0.05). No patient had any migraine attack the day following MRI.

### RS-FC

**Figure 1:**
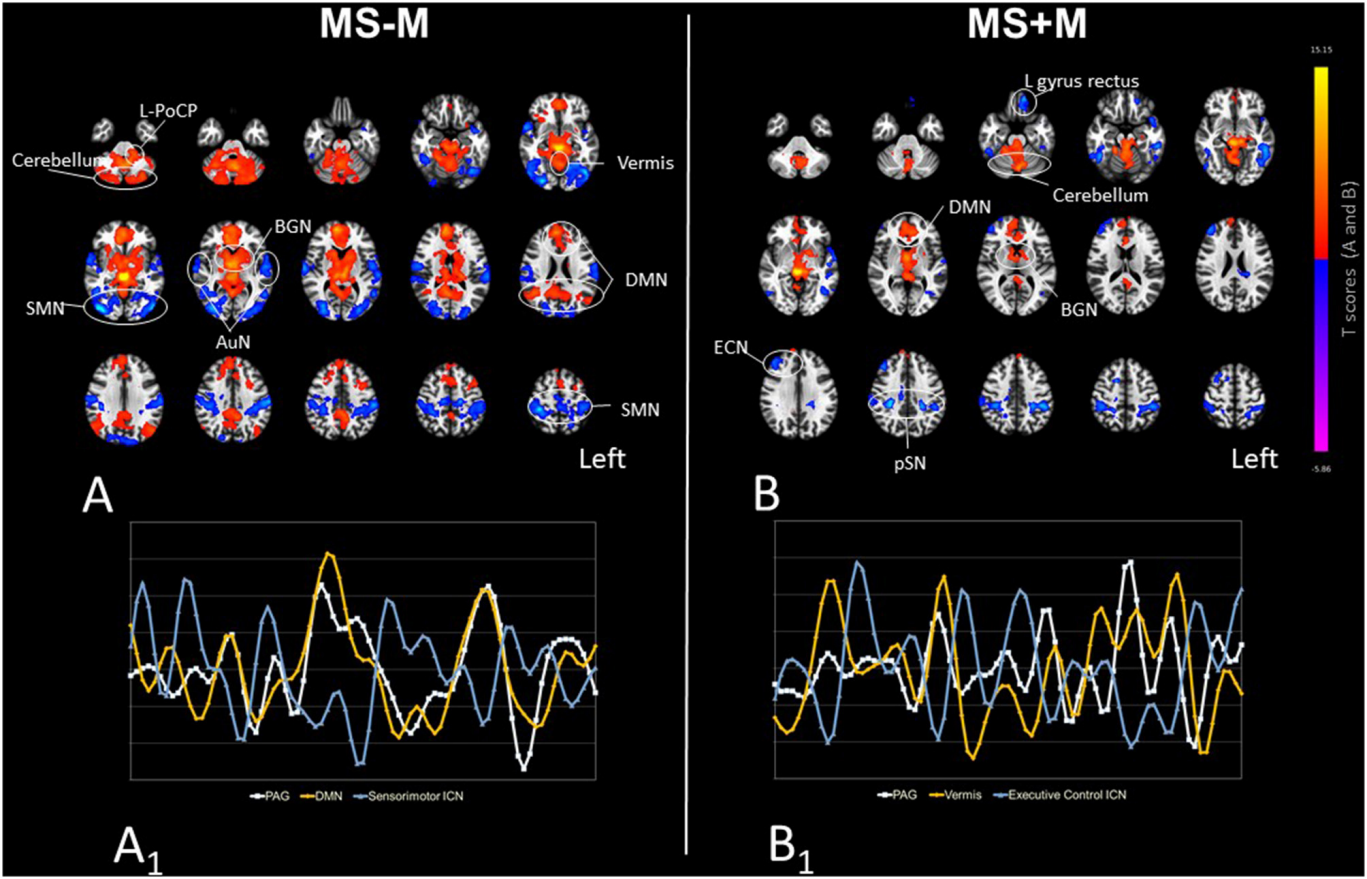
The PAG RS-FC organization in MS-M **(A)** and MS+M **(B)** patients. Maps illustrate the areas with positive (hot colors clusters) and negative (cold colors clusters) correlation with the PAG, obtained using one-sample *t*-tests and set at the statistical threshold of *P* = 0.05 after FDR correction. The PAG-positively connected brain areas represent the “pain modulatory network” (12). Images are presented in radiological convention, overlaid on a standard ICBM-152 template for anatomical reference. Connectivity cluster labels are also provided (circles), and abbreviations are defined in the main text. **(A**_**1**_**, B**_**1**_**)** illustrate exemplary time-courses of the spontaneous BOLD LFF extracted from the PAG seed, and from the areas exhibiting the strongest positive correlation (yellow) and negative correlation (blue) with the PAG, respectively in MS-M and MS+M. Negative-correlations are characterized by an out-of-phase time-course with respect to the PAG.

Head movements (along the three translation and three rotation axes) did not significantly differ between groups (FDR-corrected *P* = 0.19–0.80, two-sample *t*-test). Both groups exhibited positive and negative correlations with the PAG (*P* < 0.05, cluster-size FDR-corrected; Figure 1, Supplementary Table 1). Supplementary Figure 2 further illustrates the clusters with spatial overlap with the principal ICNs. No cluster exhibited spatial overlap with two or more different ICNs.

In MS-M patients, positive connections were present between PAG and the default mode network (DMN), and between the PAG and basal ganglia network (BGN) (*T* range = 4.01–8.02). PAG positively-correlated areas outside the main ICNs were present in the cerebellum hemisphere (*T* = 5.78), vermis (*T* = 5.96) and in the Left Posterior caudal pons (L-PoCP) (*T* = 3.46). Negative connections were present between PAG, the sensorimotor (right-SMN *T* = −6.17; left-SMN *T* = −5.57), extra-striate high visual (right-HVN *T* = −6.05; left-HVN *T* = −5.61) and auditory networks (right-AuN *T* = −4.89; left-AuN *T* = −4.62) (Supplementary Table 1, Figure 1). In MS+M patients a relatively smaller number of clusters with positive connectivity was present, specifically at the level of the vermis (*T* = 7.43), right cerebellar hemisphere (*T* = 5.58), DMN regions (*T* range = 2.9–4.43), and BGN regions (*T* range = 2.96–6.37). Negatively-correlated clusters were detected at the level of the executive control (left-ECN *T* = −5.64; right-ECN *T* = −5.08) and posterior salience (left-pSN *T* = −5.19; right-pSN *T* = −4.68) networks, in the left (*T* = −5.56) and right (*T* = −3.62) inferior temporal gyrus, and left gyrus rectus (*T* = −5.11) (Figure 1).

**Figure 2:**
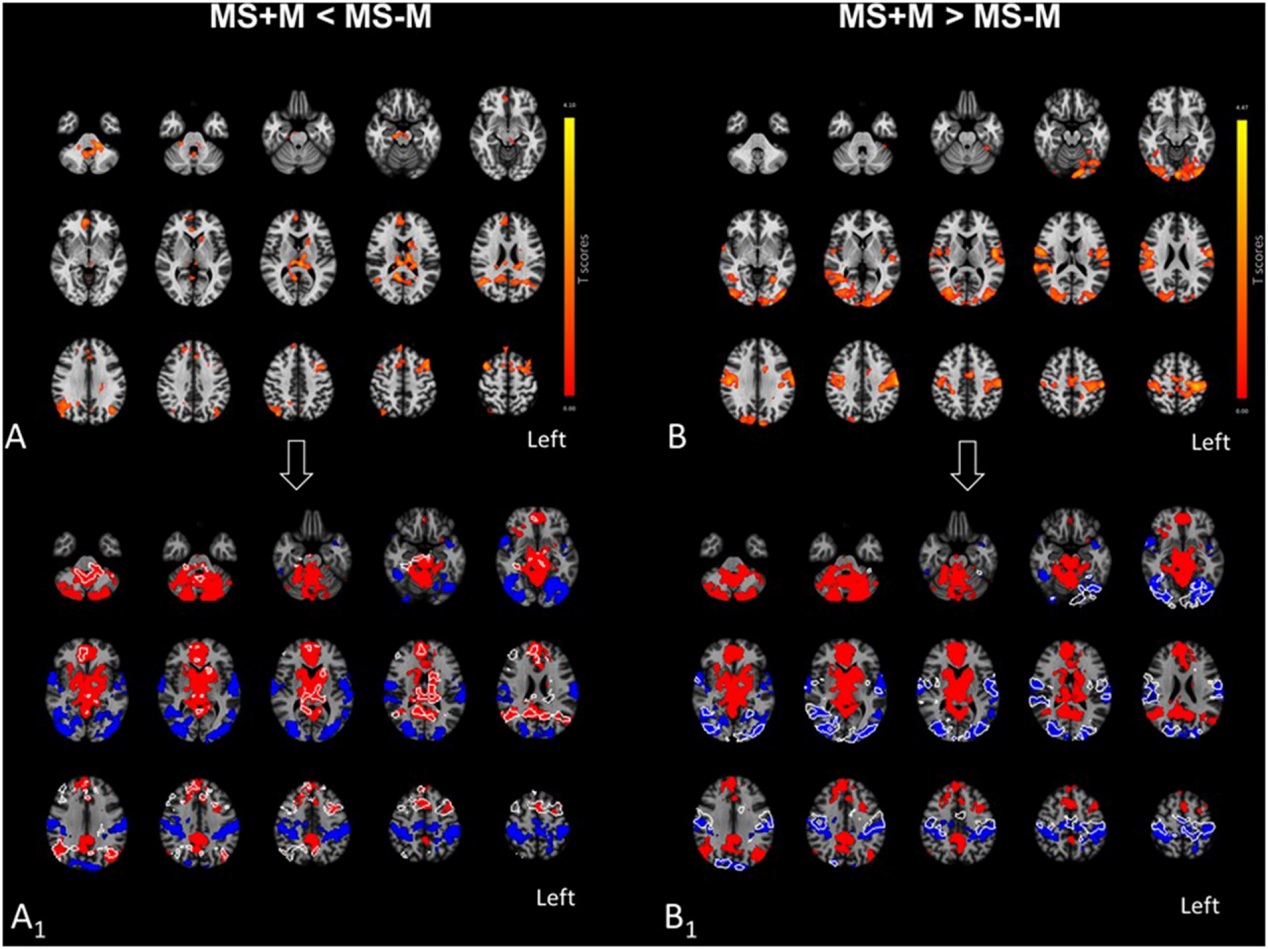
Effect of migraine on the PAG functional organization outside pain episodes, in patients with MS. In the upper panel, **(A,B)** show the areas where MS+M patients exhibited reductions and increases of PAG connectivity as compared to MS-M patients, respectively. In the lower panel, **(A**_**1**_**,B**_**1**_**)** provide white outlines of the clusters respectively shown in **(A,B)**, in comparison with the positive and negative PAG RS-FC of MS-M patients. Outlines are overlaid on the same MS-M and MS+M PAG connectivity maps of MS-M patients which were presented in Figure 1A. In **(A**_**1**_**)**, connectivity reductions in MS+M patients [outlines in **(A**_**1**_**)**] corresponded to areas where MS-M patients were *positively* connected with the PAG, thus suggesting a loss of functional integration in MS+M. In contrast, connectivity increases in MS+M [outlines in **(B**_**1**_**)**] developed in areas where MS-M patients were *negatively* connected (underlying blue clusters), possibly suggesting a loss of functional segregation with respect to MS-M. Results are presented at the statistical threshold of *P* = 0.05, FDR-corrected, in radiological convention, and overlaid on a standard MNI template for anatomical reference.

**Figure 3:**
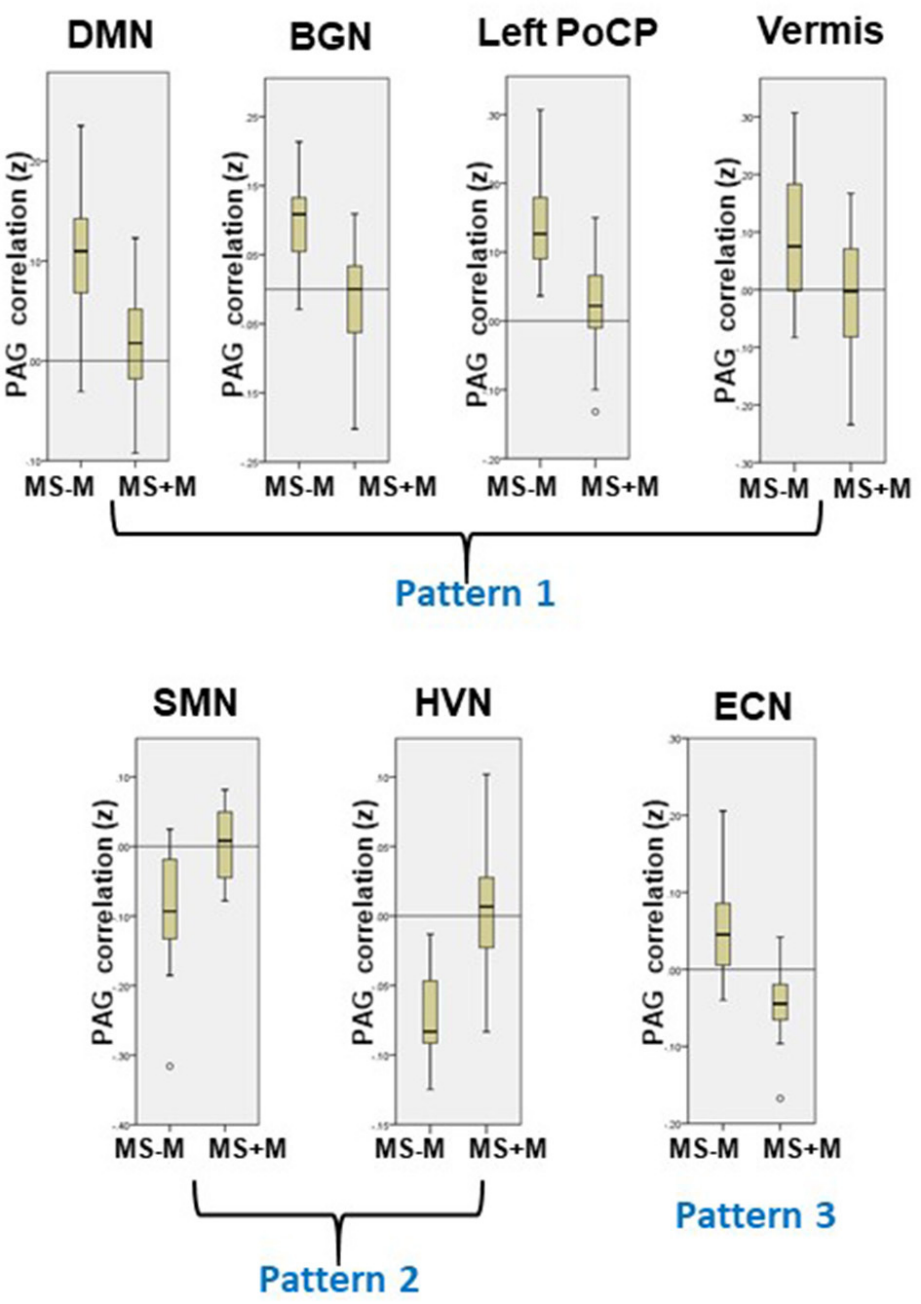
Patterns of PAG RS-FC changes in MS+M patients. Three dissociable patterns of correlation changes emerged from the PAG-based connectivity analysis (see also Table 3): Pattern 1 includes regions with PAG *positive* connectivity loss (DMN, BGN, left PoCP, and cerebellar Vermis); Pattern 2 includes regions with PAG *negative* correlation loss (SMN and HVN); Pattern 3 was characterized by loss of PAG *positive* and development of PAG *negative* connectivity (ECN). The yellow boxes show the RS-FC values distribution, for each region exhibiting significant differences with respect to MS-M (see also Figure 2).

PAG connectivity differed in several brain regions between MS+M and MS-M patients (Table 2, Figure 2). Because such variations could correspond to either RS-FC decrease or increase with respect to areas either positively or negatively connected with the PAG, results were laid over MS-M patients' PAG connectivity maps for reference. Three distinct patterns of connectivity changes emerged, and their values distribution between the groups are presented by the boxplots in Figure 3. Pattern 1 included the fROIs with PAG *positive* connectivity reduction, specifically confined to the L-PoCP, vermis, BGN, and DMN. Pattern 2 encompassed the fROIs with PAG *negative* connectivity reduction located in the SMN and HVN. Pattern 3 was characterized by inversion from positive to negative connectivity in the dorsal prefrontal areas corresponding to the ECN.

**Table 2:** Differences between MS+M and MS-M patients in the PAG connectivity organization.

	**ICN**	**Peaks**	**BA**	**Side**	**MNI coordinates**			**Peak-level *T*-value**
					**x**	**y**	**z**	
MS+M < MS-M	-	Posterior caudal pons	-	L	−17	−39	−38	−3.53
	Executive control	Middle frontal gyrus	6	L	−23	11	66	−3.52
		Middle frontal gyrus	6	R	41	7	58	−3.31
	-	Vermis	-	-	4	−61	−44	−3.38
	Basal ganglia	Caudate head	-	L	−12	7	15	−2.73
		Thalamus	-	R	10	−24	10	−2.94
		Thalamus	-	L	−9	−13	−15	−3.27
	Default mode network	Precuneus	23	R	14	−48	16	−3.18
		Angular	39	R	45	−61	26	−3.02
		Medial prefrontal cortex	9	R	12	46	24	−2.96
		Angular	39	L	−46	−69	31	−2.95
MS+M > MS-M	Sensori-motor	Postcentral gyrus	1	L	−61	−18	40	4.37
		Postcentral gyrus	1	R	34	−29	66	3.71
	Higher visual	Inferior occipital gyrus	19	L	−46	−84	−7	3.59
		Middle occipital gyrus	19	R	46	−82	−2	3.35

*Areas with spatial overlap with principal brain intrinsic connectivity networks (ICNs) (32) were grouped together. Results are presented at P <0.05 cluster size, FDR-corrected for multiple comparisons*.

*MS+M, Multiple sclerosis patients with migraine; MS-M, Multiple sclerosis patients without migraine, PAG, periaqueductal gray matter; ICNs, intrinsic connectivity networks; FDR, false discovery rate; RS-FC, resting state-functional connectivity; BA, Brodmann area; MNI, Montreal Neurological Institute*.

### Association Between Functional Changes and Migraine Symptoms

**Figure 4:**
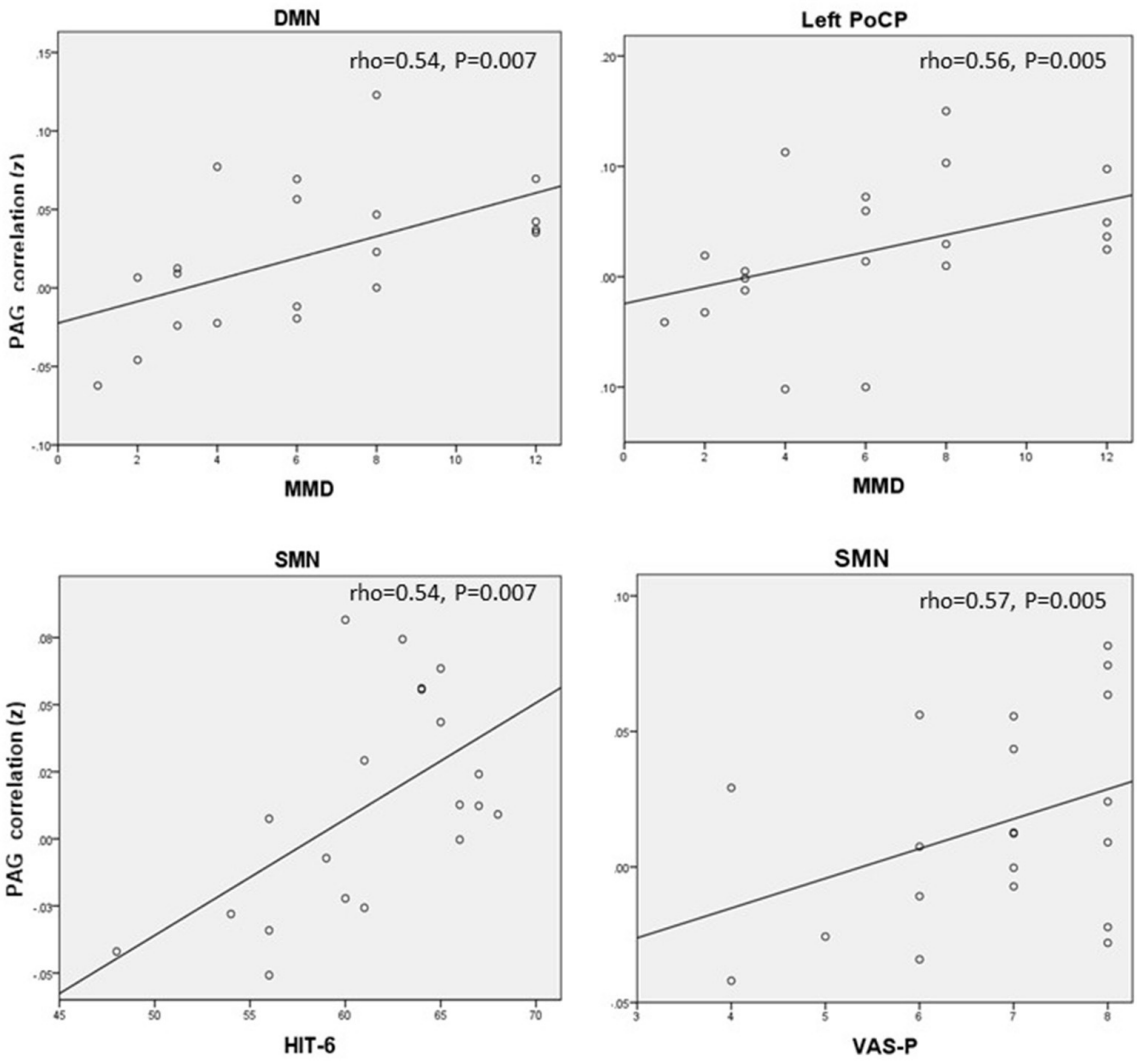
Correlations between PAG RS-FC and clinical assessment scores. MMD, Monthly migraine days; HIT-6, six-item headache impact; VAS-P, visual analog scale.

Table 3 illustrates the correlations of the PAG RS-FC with the frequency and severity of migraine attacks, and with the HIT-6 score. The corresponding values distribution are reported in Figure 4.

**Table 3:** Correlations between PAG RS-FC with migraine clinical measures scores in MS+M patients.

	**fROI**	**MMD**	**HIT-6**	**VAS-P**
		**rho (*P*)**	**rho (*P*)**	**rho (*P*)**
Pattern 1: Positive connectivity loss	L-PoCP	0.56 (0.005)^**^	−0.29 (0.104)	−0.28 (0.111)
	Default mode network	0.54 (0.007)^**^	−0.29 (0.109)	−0.32 (0.085)
	Basal ganglia ICN	−0.27 (0.128)	0.38 (0.049)^*^	0.23 (0.166)
	Vermis	−0.24 (0.156)	−0.08 (0.366)	−0.06 (0.399)
Pattern 2: Negative connectivity loss	Sensorimotor ICN	−0.46 (0.162)	0.54 (0.007)^**^	0.57 (0.005)^**^
	High visual ICN	0.014 (0.476)	0.16 (0.253)	0.23 (0.160)
Pattern 3: Negative connectivity development	Executive control ICN	−0.45 (0.023)^*^	0.12 (0.305)	0.25 (0.386)

*PAG, periaqueductal gray matter; RS-FC, resting state-functional connectivity; MS+M, Multiple sclerosis patients with migraine; fROI, functionally defined region-of-interest; MMD, Monthly migraine days; VAS-P, Visual analog scale; VAS-P, Visual analog scale; HIT-6, Six-item headache impact; L-PoCP, Left Posterior caudal pons; ICN, intrinsic connectivity network*.

* *Significant at P < 0.05;*

** *Significant at P < 0.05 after Bonferroni correction for multiple comparisons (three tests)*.

In Pattern 1 fROIs, despite the PAG DMN and L-PoCP connectivity was reduced compared to MS-M, a positive relationship was found between MMD and the L-PoCP (rho = 0.56) and between MMD and DMN (rho = 0.54), linking higher MMD to a stronger PAG connectivity. In Pattern 2 fROIs a positive correlation was observed between the SMN connectivity strength and the HIT-6 (rho = 0.54) and VAS-P scores (rho = 0.57). This reduction of negative connectivity corresponds to a more severe impact and symptoms' perception.

For Pattern 3 fROIs, inverse correlations between the ECN connectivity and MMD (rho = −0.45).

## Discussion

This study provided two main results. First, concurring with findings in healthy subjects (11, 12), the intrinsic PAG connectivity organization in MS-M was characterized by positive connectivity with brainstem, thalamic, BGN, and DMN regions, as well as negative connectivity with unimodal (sensorimotor, visual, and auditory) ICN regions, putative markers of network integration and segregation. Secondly, the occurrence of migraine in MS patients was not related to the T_2_-visible lesion load and/or spatial frequency, but was associated with the loss of *positive* and *negative* connectivity, respectively linked to the frequency and severity of the attacks.

In line with findings in healthy subjects (11, 12), in MS-M patients the PAG displayed *positive* connectivity with cortical areas belonging to the DMN, but also deep structures such as the basal ganglia and the brainstem, the so-called “PMN” (12, 33). Additionally, the PAG was *negatively* connected with unimodal sensory (i.e., sensorimotor, visual, and auditory) regions. The presence of RS-FC negative correlation (or anti-correlation) has been widely documented (13, 34, 35) and further supported by magnetoencephalography recordings of the spontaneous brain activity (36). Whereas, positive correlations are thought to reflect synchronization and integration of neuronal activity and information processing, negative correlations were hypothesized to represent segregation of neuronal activity between areas involved in competing functions (13, 14) mediated by higher level ICNs inhibitory networks (37). Our interpretation is based on previous evidence about the functional significance of negative correlations in healthy subjects (e.g., (38)) and neurological patients (e.g., (39)). In particular, a series of studies conducted in stroke patients (reviewed in (34)) have indicated that the behavioral deficits following stroke are associated with both reduced inter-hemispheric functional connectivity and reduced anti-correlation between the fronto-parietal and the default mode networks. In our study group, MS+M showed three distinct patterns of PAG connectivity reorganization between pain episodes. The most extensive rearrangement (Pattern 1) corresponded to the decreased positive RS-FC between the PAG and regions belonging to the DMN, BGN, cerebellum, and L-PoCP. Notably, the PoCP corresponds to the anatomical site of the trigeminal nucleus, found to elicit BOLD signal response to facial nociceptive stimuli (40). Thus, migraine in MS patients implicates an extensive impairment of the functional integration between the main cortical regions supporting spontaneous brain function, the DMN (31). The DMN and L-PoCP connectivity were also related to patients' MMD. Although the MS+M patients experienced a substantial decrease in the positive RS-FC from the PAG compared to MS-M patients, the residual DMN and L-PoCP connectivity strength was directly correlated to a higher MMD. Because the connectivity between DMN and L-PoCP was reduced in MS+M patients, we argue that its residual strength might represent an adaptive mechanism in response to relatively more frequent attack recurrence. However, dedicated subregional connectivity studies (such as in (41)) are needed to clarify the specific characteristics of the PAG connectivity with the trigeminal nucleus. Pattern 2 consisted of the functional segregation loss between the PAG and the SMN, and PAG and HVN. SMN and PAG are both activated during nociceptive fMRI tasks (32); however, these two regions normally show negative connectivity in healthy subjects (11, 12), a result replicated in our MS-M patients group. As migraine is known to be associated with increased excitability in the sensorimotor cortex (42, 43), we speculate that a failure to maintain interictal physiological network segregation may result in an aberrant SMN hyperexcitability within pain processing pathways.

Pattern 3 enclosed the occurrence of negative connectivity with prefrontal regions belonging to the ECN. The prefrontal cortex is considered the main source of cognitive control over pain perception (2, 44). In non-MS migraineurs, a higher MMD was indeed linked to a decreased connectivity between the PAG and several prefrontal regions, whose interictal dysfunction could promote attack precipitations (33). A similar inverse relationship between the PAG-to-ECN strength and patients' MMD was observed in our patients, although this correlation did not remain significant after adjusting for multiple comparisons.

The extended *decrease* of PAG connectivity in our MS+M patients appears different from the general *increase* of connectivity between the PAG and other regions (5, 33). This might be explained by differences in the assessment and interpretation of the negative connectivity changes. Alternatively, one may hypothesize an influence of MS-related demyelination and/or degeneration, (32) but the extent and spatial distribution frequency of demyelinating plaques, and the brain volumes, were similar between MS-M and MS+M patients.

Our study is not without limitations. Our sample size was relatively small, a fact that led to a limited statistical power. Furthermore, we did not investigate whether the functional modifications detected in our patients were specific to the MS-related migraine condition, or share similarities with otherwise healthy migraineurs. Future investigations, including patients with MS-unrelated migraine and healthy subjects, are warranted to clarify this point. In line with the demographic characteristics of the population of MS patients, our study group included more females than males. Importantly, gender-related differences were demonstrated in the RS-FC patterns of several brain areas implicated in pain processing, including the anterior cingulate cortex, the insula, the parahippocampal gyrus, the midcingulate cortex, and the temporal pole (11). Whereas, our RS-FC analyses were adjusted for gender as a covariate, future studies assessing the effect of gender are warranted to provide a specific framework of the circuitries underlying pain processing in male and female MS patients with migraine. Finally, we lack pain stimuli evoked data acquired right before, during, and immediately after nociceptive stimulation that may be useful to dynamically assess the causative relationships underlying the PAG connectivity variations.

In conclusion, we showed a breakdown of physiologically diverging patterns of PAG connectivity in MS patients with migraine, which were linked to symptoms severity and impact on daily life activities.

## Data Availability Statement

The raw data supporting the conclusions of this article will be made available by the authors, without undue reservation.

## Ethics Statement

The studies involving human participants were reviewed and approved by Local Ethics Committee reference: CE 2843. The patients/participants provided their written informed consent to participate in this study.

## Author Contributions

EP and GCR: contributed to the design of the work and to the data collection, performed the statistical analysis and interpreted data, drafted the manuscript, revised it critically for intellectual content and approved the final version of the paper. CS: performed the statistical analysis, interpreted the data and approved the final version of the paper. RS and AC: revised the manuscript for intellectual content and approved the final version of the paper. CG and CZ: contributed to conceptualization of the work, interpreted the data, supervised manuscript preparation, revised it critically for intellectual content and approved the final version of the paper.

## Conflict of Interest

The authors declare that the research was conducted in the absence of any commercial or financial relationships that could be construed as a potential conflict of interest.

## Publisher's Note

All claims expressed in this article are solely those of the authors and do not necessarily represent those of their affiliated organizations, or those of the publisher, the editors and the reviewers. Any product that may be evaluated in this article, or claim that may be made by its manufacturer, is not guaranteed or endorsed by the publisher.

## References

[1] Applebee A. The clinical overlap of multiple sclerosis and headache. Headache. (2012) 52(Suppl.2):111–6. 10.1111/j.1526-4610.2012.02243.x 23030543

[2] Tracey I Mantyh PW. The cerebral signature for pain perception and its modulation. Neuron. (2007) 55:377–91. 10.1016/j.neuron.2007.07.012 17678852

[3] Veloso F Kumar K Toth C. Headache secondary to deep brain implantation. Headache. (1998) 38:507–15. 10.1046/j.1526-4610.1998.3807507.x 15613166

[4] Reuter U McClure C Liebler E Pozo-Rosich P. Non-invasive neuromodulation for migraine and cluster headache: a systematic review of clinical trials. J Neurol Neurosurg Psychiatry. (2019) 90:796–804. 10.1136/jnnp-2018-320113 30824632 PMC6585264

[5] Schwedt TJ Vargas B. Neurostimulation for treatment of migraine and cluster headache. Pain Med. (2015) 16:1827–34. 10.1111/pme.12792 26177612 PMC4572909

[6] Gee JR Chang J Dublin AB Vijayan N. The association of brainstem lesions with migraine-like headache: an imaging study of multiple sclerosis. Headache. (2005) 45:670–7. 10.1111/j.1526-4610.2005.05136.x 15953299

[7] Tortorella P Rocca MA Colombo B Annovazzi P Comi G Filippi M. Assessment of MRI abnormalities of the brainstem from patients with migraine and multiple sclerosis. J Neurol Sci. (2006) 244:137–41. 16530789 10.1016/j.jns.2006.01.015

[8] Papadopoulou A Naegelin Y Weier K Amann M Hirsch J von Felten S. MRI characteristics of periaqueductal lesions in multiple sclerosis. Mult Scler Relat Disord. (2014) 3:542–51. 10.1016/j.msard.2014.01.001 25877067

[9] Seixas D Foley P Palace J Lima D Ramos I Tracey I. Pain in multiple sclerosis: a systematic review of neuroimaging studies. Neuroimage Clin. (2014) 5:322–31. 10.1016/j.nicl.2014.06.014 25161898 PMC4141976

[10] Fox MD Raichle ME. Spontaneous fluctuations in brain activity observed with functional magnetic resonance imaging. Nat Rev Neurosci. (2007) 8:700–11. 10.1038/nrn2201 17704812

[11] Coulombe MA Erpelding N Kucyi A Davis KD. Intrinsic functional connectivity of periaqueductal gray subregions in humans. Hum Brain Mapp. (2016) 37:1514–30. 10.1002/hbm.23117 26821847 PMC6867375

[12] Kong J Tu PC Zyloney C Su TP. Intrinsic functional connectivity of the periaqueductal gray, a resting fMRI study. Behav Brain Res. (2010) 211:215–9. 10.1016/j.bbr.2010.03.042 20347878 PMC2862838

[13] Fox MD Snyder AZ Vincent JL Corbetta M Van Essen DC Raichle ME. The human brain is intrinsically organized into dynamic, anticorrelated functional networks. Proc Natl Acad Sci USA. (2005) 102:9673–8. 10.1073/pnas.0504136102 15976020 PMC1157105

[14] Fox MD Zhang D Snyder AZ Raichle ME. The global signal and observed anticorrelated resting state brain networks. J Neurophysiol. (2009) 101:3270–83. 10.1152/jn.90777.2008 19339462 PMC2694109

[15] Pantano P Petsas N Tona F Sbardella E. The role of fMRI to assess plasticity of the motor system in MS. Front Neurol. (2015) 6:55. 10.3389/fneur.2015.00055 25852634 PMC4360702

[16] Rocca MA Pravatá E Valsasina P Radaelli M Colombo B Vacchi L. Hippocampal-DMN disconnectivity in MS is related to WM lesions and depression. Hum Brain Mapp. (2015) 36:5051–63. 10.1002/hbm.22992 26366641 PMC6869286

[17] Polman CH Reingold SC Banwell B Clanet M Cohen JA Filippi M. Diagnostic criteria for multiple sclerosis: 2010 revisions to the McDonald criteria. Ann Neurol. (2011) 69:292–302. 10.1002/ana.22366 21387374 PMC3084507

[18] Schiffman E Ohrbach R Truelove E Look L Anderson G Goulet J-P. The international classification of headache disorders, 3rd edition (beta version). Cephalalgia. (2013) 33:629–808. 10.1177/0333102413485658 23771276

[19] Kosinski M Bayliss MS Bjorner JB Ware JE Garber WH Batenhorst A. A six-item short-form survey for measuring headache impact: the HIT-6. Qual Life Res. (2003) 12:963–74. 10.1023/A:1026119331193 14651415

[20] Hamilton M. A rating scale for depression. J Neurol Neurosurg Psychiatry. (1960) 23:56–62. 10.1136/jnnp.23.1.56 14399272 PMC495331

[21] Hamilton M. The assessment of anxiety states by rating. Br J Med Psychol. (1959) 32:50–5. 10.1111/j.2044-8341.1959.tb00467.x 13638508

[22] Kaza E Klose U Lotze M. Comparison of a 32-channel with a 12-channel head coil: are there relevant improvements for functional imaging? J Magn Reson Imaging. (2011) 34:173–83. 10.1002/jmri.22614 21618334

[23] Rorden C Karnath HO Bonilha L. Improving lesion-symptom mapping. J Cogn Neurosci. (2007) 19:1081–8. 10.1162/jocn.2007.19.7.1081 17583985

[24] Whitfield-Gabrieli S Nieto-Castanon A. Conn: a functional connectivity toolbox for correlated and anticorrelated brain networks. Brain Connect. (2012) 2:125–41. 10.1089/brain.2012.0073 22642651

[25] Chai XJ Castañón AN Ongür D Whitfield-Gabrieli S. Anticorrelations in resting state networks without global signal regression. Neuroimage. (2012) 59:1420–8. 10.1016/j.neuroimage.2011.08.048 21889994 PMC3230748

[26] Behzadi Y Restom K Liau J Liu TT. A component based noise correction method (CompCor) for BOLD and perfusion based fMRI. Neuroimage. (2007) 37:90–101. 10.1016/j.neuroimage.2007.04.042 17560126 PMC2214855

[27] Ezra M Faull OK Jbabdi S Pattinson KT. Connectivity-based segmentation of the periaqueductal gray matter in human with brainstem optimized diffusion MRI. Hum Brain Mapp. (2015) 36:3459–71. 10.1002/hbm.22855 26138504 PMC4755135

[28] Linnman C Moulton EA Barmettler G Becerra L Borsook D. Neuroimaging of the periaqueductal gray: state of the field. Neuroimage. (2012) 60:505–22. 10.1016/j.neuroimage.2011.11.095 22197740 PMC3288184

[29] Chen G Padmala S Chen Y Taylor PA Cox RW Pessoa L. To pool or not to pool: Can we ignore cross-trial variability in FMRI? Neuroimage. (2021) 225:117496. 10.1016/j.neuroimage.2020.117496 33181352 PMC7861143

[30] Zarifkar P Kim J La C Zhang K YorkWilliams S Levine TF. Cognitive impairment in Parkinson's disease is associated with Default Mode Network subsystem connectivity and cerebrospinal fluid A?. Parkinsonism Relat Disord. (2021) 83:71–8. 10.1016/j.parkreldis.2021.01.002 33484978 PMC7940579

[31] Shirer WR Ryali S Rykhlevskaia E Menon V Greicius MD. Decoding subject-driven cognitive states with whole-brain connectivity patterns. Cereb Cortex. (2012) 22:158–65. 10.1093/cercor/bhr099 21616982 PMC3236795

[32] Moisset X Ouchchane L Guy N Bayle DJ Dallel R Clavelou P. Migraine headaches and pain with neuropathic characteristics: comorbid conditions in patients with multiple sclerosis. Pain. (2013) 154:2691–9. 10.1016/j.pain.2013.07.050 23911697

[33] Mainero C Boshyan J Hadjikhani N. Altered functional magnetic resonance imaging resting-state connectivity in periaqueductal gray networks in migraine. Ann Neurol. (2011) 70:838–45. 10.1002/ana.22537 22162064 PMC3243965

[34] Baldassarre A Ramsey LE Siegel JS Shulman GL Corbetta M. Brain connectivity and neurological disorders after stroke. Curr Opin Neurol. (2016) 29:706–13. 10.1097/WCO.0000000000000396 27749394 PMC5682022

[35] Chang C Glover GH. Time-frequency dynamics of resting-state brain connectivity measured with fMRI. Neuroimage. (2010) 50:81–98. 10.1016/j.neuroimage.2009.12.011 20006716 PMC2827259

[36] Baker AP Brookes MJ Rezek IA Smith SM Behrens T Probert Smith PJ. Fast transient networks in spontaneous human brain activity. Elife. (2014) 3:e01867. 10.7554/eLife.01867 24668169 PMC3965210

[37] Zhou Y Friston KJ Zeidman P Chen J Li S Razi A. The hierarchical organization of the default, dorsal attention and salience networks in adolescents and young adults. Cerebral Cortex. (2018) 28:726–37. 10.1093/cercor/bhx307 29161362 PMC5929108

[38] Kelly AM Uddin LQ Biswal BB Castellanos FX Milham MP. Competition between functional brain networks mediates behavioral variability. Neuroimage. (2008) 39:527–37. 10.1016/j.neuroimage.2007.08.008 17919929

[39] Baldassarre A Ramsey L Hacker CL Callejas A Astafiev SV Metcalf NV. Large-scale changes in network interactions as a physiological signature of spatial neglect. Brain. (2014) 137(Pt 12):3267–83. 10.1093/brain/awu297 25367028 PMC4240302

[40] DaSilva AF Becerra L Makris N Strassman AM Gonzalez RG Geatrakis N. Somatotopic activation in the human trigeminal pain pathway. J Neurosci. (2002) 22:8183–92. 10.1523/JNEUROSCI.22-18-08183.2002 12223572 PMC6758094

[41] Schulte LH May A. The migraine generator revisited: continuous scanning of the migraine cycle over 30 days and three spontaneous attacks. Brain. (2016) 139(Pt 7):1987–93. 10.1093/brain/aww097 27190019

[42] Aurora SK Wilkinson F. The brain is hyperexcitable in migraine. Cephalalgia. (2007) 27:1442–53. 10.1111/j.1468-2982.2007.01502.x 18034688

[43] Lang E Kaltenhäuser M Neundörfer B Seidler S. Hyperexcitability of the primary somatosensory cortex in migraine–a magnetoencephalographic study. Brain. (2004) 127:2459–69. 10.1093/brain/awh295 15471903

[44] Lorenz J Minoshima S Casey KL. Keeping pain out of mind: the role of the dorsolateral prefrontal cortex in pain modulation. Brain. (2003) 126:1079–91. 10.1093/brain/awg102 12690048

