# Supplementary figures and images for: Migraine in Multiple Sclerosis Patients Affects Functional Connectivity of the Brain Circuitry Involved in Pain Processing
Source: Front Neurol. 2021 Aug 12;12:690300. doi: 10.3389/fneur.2021.690300 (PMC8397382; doi:10.3389/fneur.2021.690300)

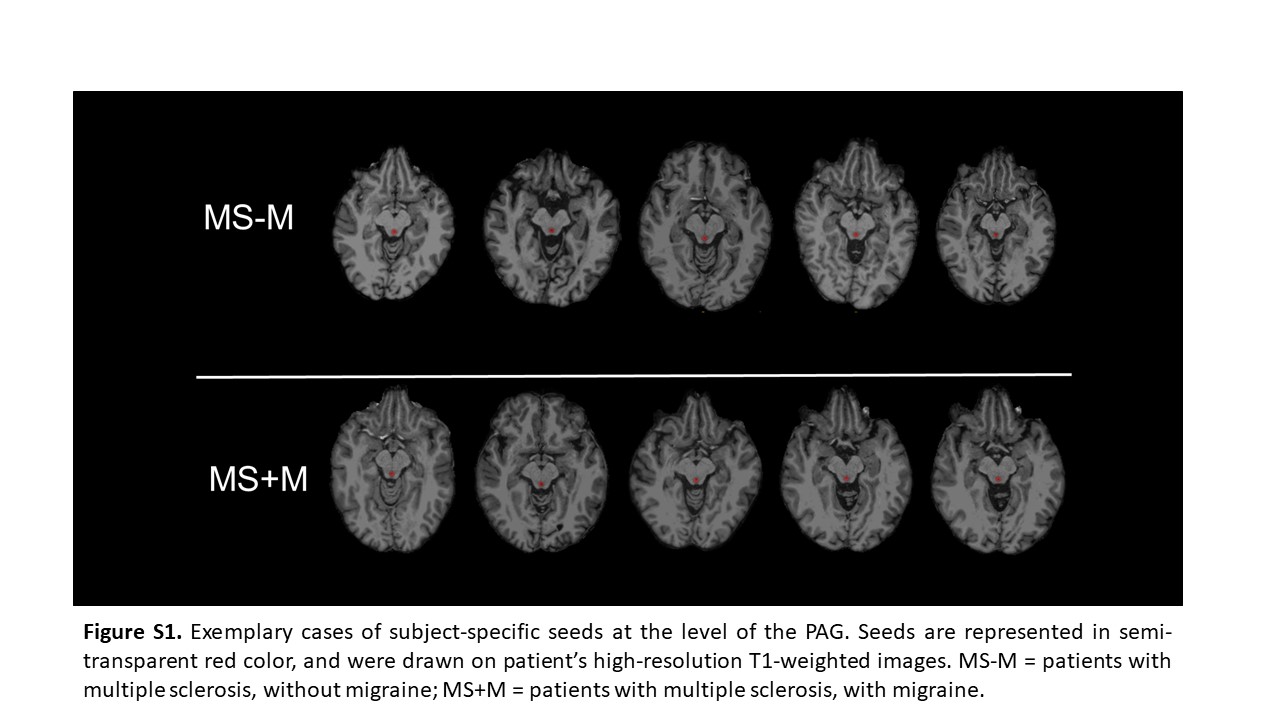

Supplement: Supplementary file 1 [file Image_1.JPEG]

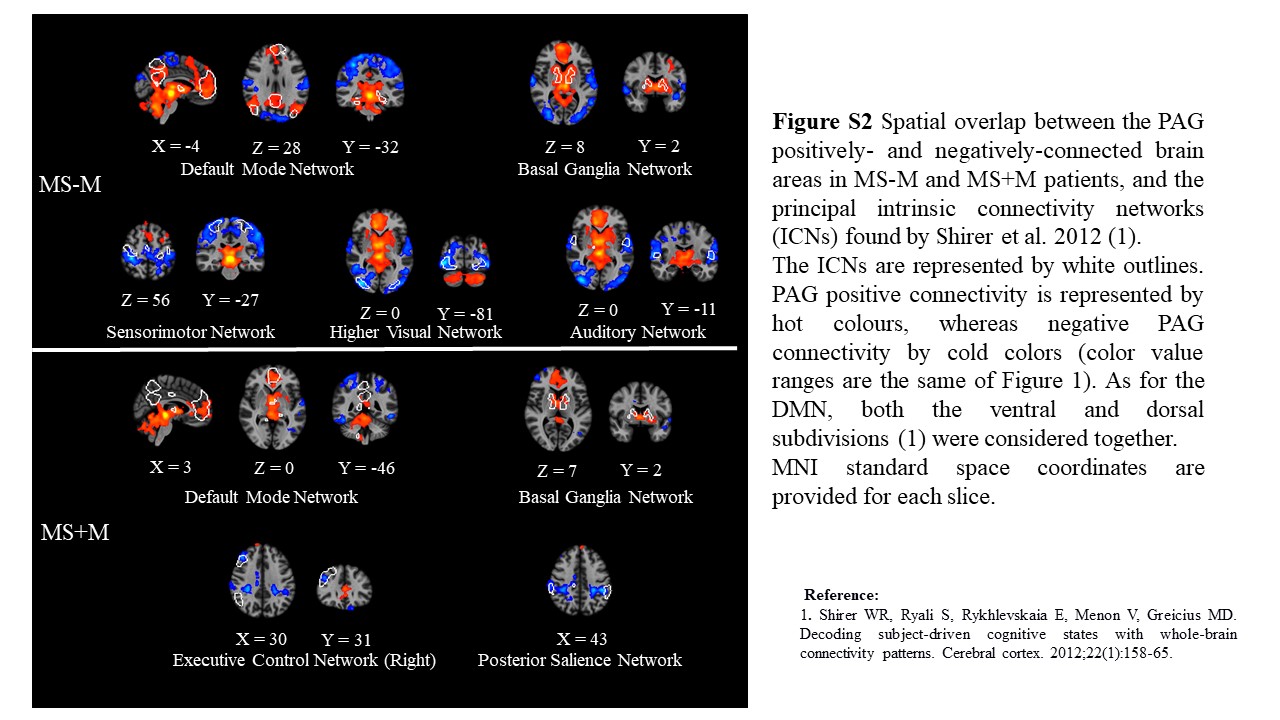

Supplement: Supplementary file 2 [file Image_2.JPEG]
