# Supplementary material for: Migraine in Multiple Sclerosis Patients Affects Functional Connectivity of the Brain Circuitry Involved in Pain Processing
Source: Front Neurol. 2021 Aug 12;12:690300. doi: 10.3389/fneur.2021.690300 (PMC8397382; doi:10.3389/fneur.2021.690300)
Supplement: Supplementary file 3 [file Table_1.docx]

**Table S1.**Brain areas with significant positive and negative periaqueductal gray mattercharacteristics inmultiple sclerosis patients without and with migraine.

| **Groups** | **Connectivity** | **ICN** | **Peaks** | | **BA** | **x** | | **y** | | **z** | | **T value**  **(peak level)** | | |
| --- | --- | --- | --- | --- | --- | --- | --- | --- | --- | --- | --- | --- | --- | --- |
|  |  |  |  |  |  | **(R / L)** | | **(R / L)** | | **(R / L)** | | **R** | | **L** |
| **MS-M** | **Positive** | Default mode network | | Medial prefrontal cortex | 10 | 11 / -10 | 55 / 57 | | 11 / 8 | | 8.02 | | 4.01 | |
|  |  |  |  | Precuneus | 23 | 4 / - | 57 / - | | 16 /- | | 5.53 | | - | |
|  |  |  |  | Angular | 39 | 45/- | -57 | | 24 | | 4.98 | | - | |
|  |  |  |  | Fusiform | 37 | - / -26 | -36 | | -17 | | - | | 6.26 | |
|  |  | - | | Cerebellar hemisphere | - | - / -36 | -80 | | -36 | | - | | 5.78 | |
|  |  |  |  | Vermis | - | - / -2 | -63 | | -37 | | - | | 5.96 | |
|  |  | Basal ganglia | | Thalamus | - | 6 / -8 | -24 / -16 | | 6 / 10 | | 5.68 | | 7.25 | |
|  |  |  |  | Caudate head | - | 13 / - | -/11 | | -/10 | | 4.48 | | - | |
|  |  |  |  | Putamen | - | - / -29 | -/5 | | -/0 | | - | | 4.40 | |
|  |  | - | | Posterior caudal pons | - | - / -13 | -37 | | -38 | | - | | 3.46 | |
|  | **Negative** | Sensorimotor | | Postcentral gyrus | 1 | 35 / -63 | -34 / -20 | | 55 / 34 | | -6.17 | | -5.57 | |
|  |  | High Visual | | Middle occipital gyrus | 19 | 43 / -45 | -85 / -83 | | 0 / -2 | | -6.05 | | -5.61 | |
|  |  | Auditory | | Superior temporal gyrus | 41 | 66 / -55 | -12 / -11 | | 8 / 3 | | -4.89 | | -4.62 | |
| **MS+M** | **Positive** | - | | Vermis | - | - /-3 | -75 | | -27 | | - | | 7.43 | |
|  |  |  |  | Cerebellar Hemisphere | - | 19 / - | -41 | | -21 | | 5.58 | | - | |
|  |  | Default mode network | | Medial prefrontal cortex |  | -8 / 7 | 43 / 52 | | 0 / -1 | | 4.43 | | 4.07 | |
|  |  |  |  | Precuneus | 23 | - /-4 | -51 | | 15 | | - | | 2.90 | |
|  |  | Basal ganglia | | Thalamus | - | 3 / -2 | -6 / 5 | | 1 / 1 | | 6.37 | | 5.52 | |
|  |  |  |  | Caudate head | - | 9 / -7 | 8 / 10 | | 9 / 1 | | 3.30 | | 2.96 | |
|  | **Negative** | Executive Control | | Middle frontal gyrus | 6 | -10 / 14 | 13 / 8 | | 66 / 54 | | -5.08 | | -5.64 | |
|  |  | Posterior Salience | | Supramarginal gyrus | 40 | -36 / 38 | -37 / -37 | | 41 / 46 | | -4.68 | | -5.16 | |
|  |  | - | | Inferior temporal gyrus | 21 | -55 / 54 | -37 / -66 | | -15 / -6 | | -3.62 | | -5.56 | |
|  |  | - | | Gyrus rectus | 11 | - / -13 | - / 53 | | - / -23 | | - | | -5.11 | |

Areas belonging to the same ICN were grouped together. The thalamus was included in the basal ganglia ICN.Cluster peaks are presented at P<0.05, FDR-corrected for multiple comparisons.

Abbreviations: BA=Brodmann area; FDR=false discovery rate; ICN=intrinsic connectivity network; L=left; MS-M=multiple sclerosis patients withoutmigraine; MS+M=multiple sclerosis patients with migraine; R=right.
